# Inhibiting tumorigenic potential by restoration of p16 in nasopharyngeal carcinoma

**DOI:** 10.1038/sj.bjc.6690818

**Published:** 1999-12

**Authors:** G L Wang, K W Lo, K S Tsang, N Y F Chung, Y S Tsang, S T Cheung, J C K Lee, D P Huang

**Affiliations:** Department of Anatomical and Cellular Pathology, Prince of Wales Hospital, The Chinese University of Hong Kong, Shatin, Hong Kong

**Keywords:** nasopharyngeal carcinoma, *p16*, tumour suppressor, gene transfer

## Abstract

The *p16* gene, encodes a key checkpoint protein p16 in the cell cycle, has been reported inactivation in a wide variety of human cancers. We have previously demonstrated high frequency of *p16* alterations in primary nasopharyngeal carcinoma (NPC), xenografts and cell lines. The finding implied that inactivation of the *p16* gene may play an important role in the NPC development. To investigate the tumour suppressor function of *p16* in NPC, we tranfected *p16*-deficient NPC cell line, NPC/HK-1, with a wild-type *p16* expression construct, and evaluated growth and tumorigenic properties of the clones stably expressing exogenous *p16*. Expression of the exogenous wild-type *p16* significantly inhibited cell growth by more than 70% when compared to that of the parental and empty vector-transfected cells. This growth inhibition was attributable to a significant proportion of p16-expressing cells arrested at G1 phase in the cell cycle as revealed by flow cytometric analysis. By anchorage-independent colony forming assay, we found that the ability to form colonies in soft agar was highly reduced in cells expressing p16. NPC/HK1 cells expressing functional p16 also showed suppressed tumorigenicity in athymic nude mice. Taken together, our results provide strong evidence for a tumour suppressor role of *p16* in NPC. © 1999 Cancer Research Campaign


*British Journal of Cancer* (1999) **81**(7), 1122–1126
© 1999 Cancer Research Campaign
Article no. bjoc.1999.0818

### Inhibiting tumorigenic potential by restoration of p16 in nasopharyngeal carcinoma

**GL Wang, KW Lo, KS Tsang, NYF Chung, YS Tsang, ST Cheung, JCK Lee and DP Huang**

Department of Anatomical and Cellular Pathology, Prince of Wales Hospital, The Chinese University of Hong Kong, Shatin, Hong Kong

**Summary** The *p16* gene, encodes a key checkpoint protein p16 in the cell cycle, has been reported inactivation in a wide variety of human cancers. We have previously demonstrated high frequency of *p16* alterations in primary nasopharyngeal carcinoma (NPC), xenografts and cell lines. The finding implied that inactivation of the *p16* gene may play an important role in the NPC development. To investigate the tumour suppressor function of *p16* in NPC, we tranfected *p16*-deficient NPC cell line, NPC/HK-1, with a wild-type *p16* expression construct, and evaluated growth and tumorigenic properties of the clones stably expressing exogenous *p16*. Expression of the exogenous wild-type *p16* significantly inhibited cell growth by more than 70% when compared to that of the parental and empty vector-transfected cells. This growth inhibition was attributable to a significant proportion of p16-expressing cells arrested at G1 phase in the cell cycle as revealed by flow cytometric analysis. By anchorage-independent colony forming assay, we found that the ability to form colonies in soft agar was highly reduced in cells expressing p16. NPC/HK1 cells expressing functional p16 also showed suppressed tumorigenicity in athymic nude mice. Taken together, our results provide strong evidence for a tumour suppressor role of *p16* in NPC. © 1999 Cancer Research Campaign

**Keywords**: nasopharyngeal carcinoma; *p16*; tumour suppressor; gene transfer

Like most solid tumours, the tumorigenesis of nasopharyngeal carcinoma (NPC) involves accumulation of multiple genetic alterations. Overexpression of several proto-oncogenes *Bcl-2*, *c-Myc* and *Ras* has been reported in this tumour (Lu et al, 1993; Porter et al, 1994). Our previous studies demonstrated that frequent allelic losses at chromosomes 3p, 9p, 11q and 14q were found in NPC(Huang, 1991, 1994; Hui, 1996; Cheng et al, 1997). Homozygous deletion in 9p21–22 has been detected both in NPC primary tumours and xenografts (Huang et al, 1994; Lo et al, 1995). Recent studies have localized three *INK4* family genes, *p16* (CNKN2A, MTS1, INK4A), *p15* (CNKN2B, INK4B) and *p19* (ARF) to this affected region (Kamb et al 1994; Hannon and Beach, 1994; Nobori et al 1994; Chan et al, 1995). Among these genes, high incidence of *p16* gene alterations (77.1%), either due to homozygous deletion or aberrant methylation resulting in lose of p16 expression, has been observed in the primary tumours (Lo, 1995; Lo et al, 1996). It is likely that *p16* is the primary target for inactivation in this region. Frequent absence of the p16 protein has also been reported (Gulley et al, 1998). Detection of high frequency of altered *p16* gene in NPC stands it out as the most common genetic abnormality found in this cancer.

The progression of a normal cell from G1 into S phase in a cell cycle is regulated by the cyclin-dependent kinases (cdk) 4/6 and cyclin D1 complex through phosphorylation of the retinoblastoma protein (pRb) at the late G1 phase (Hinds et al, 1992; Kato et al, 1993). By competing with cyclin D1 for binding to cdk4/6, the p16 protein inhibits phosphorylation of pRb and arrests cells at the G1 phase. Inactivation of *p16* may lead to persistent pRb phosphorylation and, therefore, resulting uncontrolled cell proliferation.

*Received 29 January 1999*
*Accepted 3 June 1999*

*Correspondence to:* KW Lo

The current study aimed to investigate the tumour suppressor role of *p16* in NPC. We introduced a wild-type *p16* cDNA expression construct into a *p16*-deficient NPC cell line NPC/HK-1 and examined for growth and tumorigenic parameters of the resultant transfected cells. Our data demonstrated that restoration of *p16* expression in NPC/HK1 cells suppressed growth by arresting cells at G1 phase and inhibited tumorigenicity in athymic nude mice.

### MATERIALS AND METHODS

### Cell line and transfection

The human cell line NPC/HK-1 was derived from a well-differentiated NPC tumour (Huang et al, 1980). No p16 protein is found in this cell line as one allele of the *p16* gene is deleted and the other shows mutation at the splice site of exon 2 (Lo et al, 1995). NPC/HK-1 also expresses a mutant p53 (Spruck et al, 1992) and a functional pRb. HeLa cells with intact *p16* was used as positive control. Cells were grown in RPMI-1640 medium (Sigma Chemical Company, St Louis, MO, USA) supplemented with 10% fetal bovine serum, 1% glutamine and 1% penicillin–streptomycin (Gibco-BRL, Grand Island, NY, USA).

The pCMV-p16 plasmid contains a full-length wild-type *p16* gene inserted downstream of a CMV promotor and a neomycin resistance selectable marker (Liggett et al, 1996; a gift from Professor David Sidransky, Johns Hopkins University, USA). The pCMV empty vector and the pCMV-p16 construct were transfected into NPC/HK-1 cells and HeLa cells separately using Lipofectamine reagent (Gibco-BRL, Grand Island, NY, USA) according to manufacturer's protocol. Transfected cells were selected in 300 μg $ml^{-1}$ G418 (Gibco-BRL, Grand Island, NY, USA) for 4–5 weeks. Individual G418-resistant colonies were then picked using the trypsin-soaked filter paper discs method and expanded for further analyses.

### Polymerase chain reaction

In order to detect the presence of pCMV-p16 in the transfected cells, the primers S9 and S13 flanking exon 1 to 3 of the *p16* gene were used to amplify the exogenous *p16* cDNA sequence as described (Lo et al, 1996).

### Northern blot analysis

Total RNA was isolated from cells using TRIzol reagent (Gibco-BRL, Grand Island, NY, USA) according to manufacturer's instruction. Twenty micrograms of total RNA was subjected to electrophoresis in a 1.5% formaldehyde-containing denaturing agarose gel followed by capillary transfer onto the Hybond-N membrane (Amersham, Little Chalfont, UK). A full-length p16 cDNA probe was labelled with $\alpha$-$^{32}$P[dCTP] using the Rediprime DNA labelling system (Amersham, Little Chalfont, UK). The blot was hybridized with the p16 probe using Rapid Hyb buffer (Amersham, Little Chalfont, UK). After stringency washing, the blot was exposed to Kodak X-OMAT K film (Kodak, Rochester, NY, USA). To normalize the levels of transcripts, the blot was stripped and rehybridized with a glyceraldehyde 3-phosphate dehydrogenease (GAPDH) probe.

### Western blot analysis

Protein extracts were prepared according to Pagano et al (1993). Fifty micrograms of protein were separated on a 15% sodium dodecyl sulphate polyacrylamide gel electrophoresis (SDS-PAGE) gel and electro-transferred onto the ECL-NC membrane (Amersham, Little Chalfont, UK). The blots were blocked with phosphate-buffered saline (PBS) containing 5% non-fat milk and 0.1% Tween-20 for 30 min at room temperature and treated with rabbit anti-human p16 polyclonal antibody (1:1500; PharMingen, San Diego, CA, USA) for 2 h at room temperature. After PBS wash, the blots were incubated with a peroxidase-conjugated anti-rabbit secondary antibody (1:2000; Dako, Kyoto, Japan) for 1 h. Antigen-antibody complexes were detected by the chemiluminescence reagent (Amersham, Little Chalfont, UK). The expression of Rb and cyclin D1 were detected by human monoclonal antibodies of Rb (G3-245, PharMingen, San Diego, CA, USA) and cyclin D1 (G124-326, PharMingen, San Diego, CA, USA) respectively. To ensure equal loading of protein extracts, a parallel gel was stained with Coomassie brilliant blue G250 (Sigma Chemical Company, St Louis, MO, USA).

### Cell growth and cell cycle analyses

Growth curves were constructed by cell counting over a set period of cultivation. Cells were seeded onto a 24-well plate at $2 \times 10^4$ cells per well and grew in medium. A total of 300 $\mu$g ml$^{-1}$ G418 were added to the medium for the transfected cells. Culture medium was changed every 2 days and the number of cells was counted consecutively for 7 days. Each experiment was done in triplicate.

For flow cytometry analysis, cells ($\sim 5 \times 10^6$) were harvested by trypsinization, washed with Hank's balanced salt solution (HBSS, Sigma Chemical Company, St Louis, MO, USA), fixed in 70% ethanol at 4°C for 2 h, then allowed to pass through 40 $\mu$m cell strainer (Falcon, Becton Dickinson, USA). After washing with HBSS and adjusting the cell number to $1 \times 10^6$ ml$^{-1}$ in HBSS supplying with propidium iodide (50 $\mu$g ml$^{-1}$), the cellular DNA content was assessed by the System II software in a Coulter EPICS XL MCL flow cytometer (Coulter Corporation, Miama, FL, USA) and the cell cycle distribution data were analysed by the Multicycle software (Phoenix Flow Systems, San Diego, CA, USA).

### Anchorage-independent growth assay

Five hundred exponentially growing cells were suspended in 2 ml medium containing 0.35% low-melting temperature (LMT) agarose gel (FMC, Rockland, ME, USA) and overlaid onto a layer of 2 ml 0.6% LMT agarose gel/RPMI-1640 of each well of a 6-well plate. For the p16-transfected cells, the agarose gel/medium containing 300 $\mu$g ml$^{-1}$ G418 was used. After 3 weeks, colonies composed of at least 16 cells were counted under an inverted microscope.

### Tumorigenicity

Balb/c nude mice of 7- to 8-week-old were used in the tumorigenicity assay. Cells ($3 \times 10^6$) suspended in 0.5 ml RPMI-1640 medium were injected subcutaneously into the back of each nude mouse. Tumour growth was monitored by measuring the size of tumours using a caliper.

### RESULTS

### Expression of exogenous wild-type p16

We transfected a full-length wild-type *p16* expression cassette into *p16*-null NPC/HK-1 cells and isolated colonies that were resistant to G418. Polymerase chain reaction (PCR) analysis demonstrated that six clones (N21, N24, N50, N52, N55, N104) contained the *p16* cDNA fragment, which was absent from parental cells and cells transfected with empty vector (Figure 1A). Northern blot analysis revealed the presence of a hybridizing band in these six *p16*-transfected clones, whereas parental cells showed no hybridization signal to the *p16* probe (Figure 1B). The size of hybridizing band of the exogenous *p16* transcript was greater than that of the endogenous p16 transcript, as revealed in HeLa and HeLa *p16*-transfected cells. In addition to the band corresponded to the endogenous p16 transcript, the larger hybridizing band of the exogenous *p16* transcript showed the same size as those bands seen in *p16*-transfected NPC/HK-1 cells. After normalization with the GAPDH signals, we found the expression levels of exogenous *p16* transcripts varied among the six *p16*-transfected NPC/HK-1 clones. Clone N21 expressed the highest level of *p16* transcripts. Western blot analysis was then performed to assess the expression of p16 protein. Using a p16-specific antibody, we detected a band of 16 kDa, characteristics of human p16, in protein extracts of the six clones (Figure 1C). In contrast, parental and empty vector-transfected cells showed the absence of p16 expression. Moreover, the amount of p16 protein expressed by the *p16*-transfected clones correlated with the levels of *p16* transcripts. Clone N21 with the highest expression of p16 was selected for growth and tumorigenic assays. Expressions of the pRb and cyclin D1 proteins were detected in both *p16*-transfected and parental cells (data not shown).

© 1999 Cancer Research Campaign

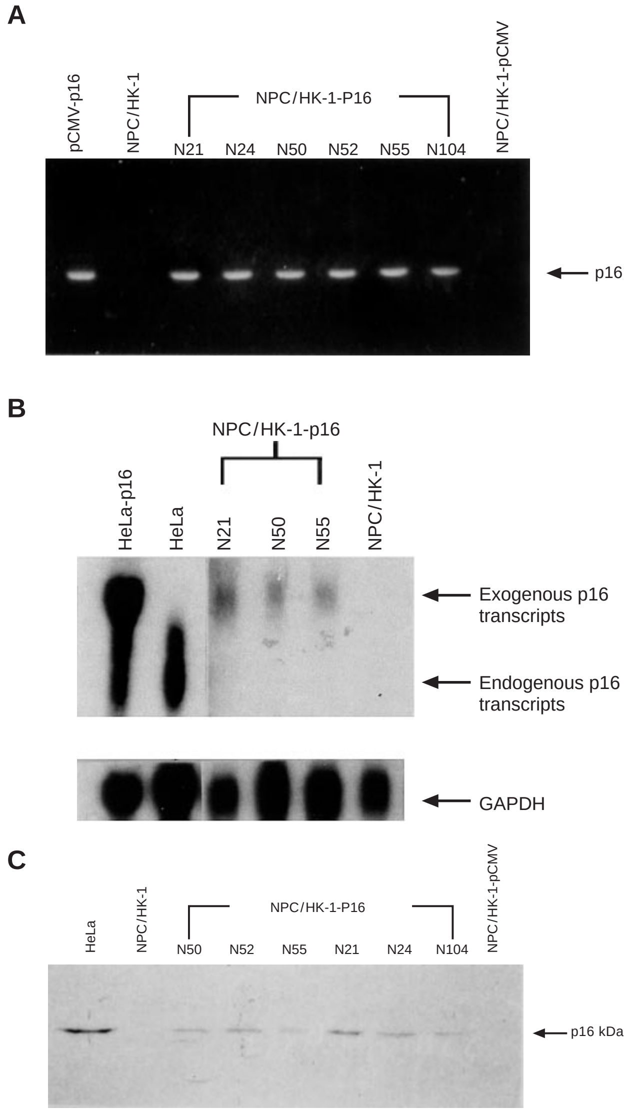

**Figure 1** Expression of exogenous *p16* in *p16*-null NPC/HK-1 cells. (**A**) Demonstration of the presence of p16 cDNA fragment in transfected cells by PCR. The expected size of the PCR product was 485 bp. (**B**) Northern blot analysis of the *p16* expression. Exogenous *p16* transcripts were detected in cells transfected with *p16*. (**C**) Western blot analysis of p16 protein expression. Clones that expressed exogenous p16 transcripts also expressed the *p16* protein

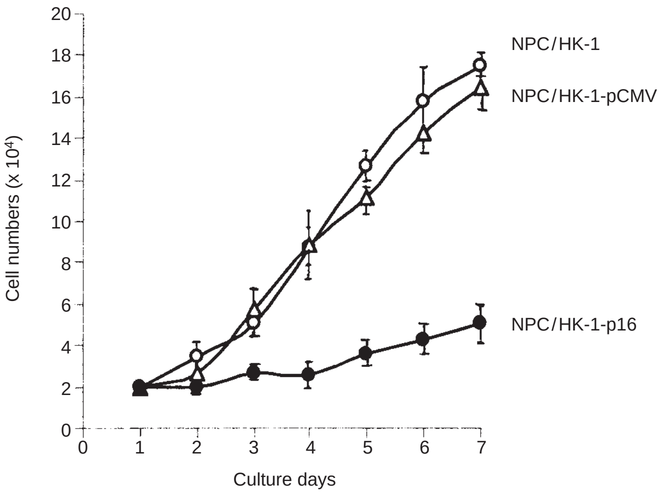

**Figure 2** Growth curves of NPC/HK-1 cells. The mean of cell counts from three independent experiments were plotted. Cell growth of p16-expressing N21 cells was reduced by 70% as compared to those of parental and empty vector-transfected cells.

**Table 1** Anchorage-independent growth in vitro and tumorigenicity in athymic nude mice by functional restoration of p16 expression in NPC/HK-1 cells

| | Anchorage-independent growth | Tumorigenicity | |
|---|---|---|---|
| Cell type | No. of colony | Nude mice | Tumour volume (mm$^3$)[a] |
| Parental | 34 | A | 530 |
| | | B | 1600 |
| Empty vector-transfected | 32 | A | 800 |
| | | B | 1200 |
| p16-transfected | 3 | A | 0[b] |
| | | B | 0[b] |

[a]Tumour size was measured 1 month post-inoculation. [b]Tumour was examined over a 10-month interval.

### Effect of p16 on cell growth

To evaluate the effects of wild-type *p16* expression on growth, we examined the growth rates of the *p16*-transfected clone N21, parental NPC/HK-1 cells and the vector-transfected cells by cell proliferation assay. There was no significant difference in the growth rate between NPC/HK-1 parental and empty vector-transfected cells (Figure 2). However, the proliferate ability of clone N21 was reduced and these cells grew at a slower rate. On day 7, growth of the *p16*-transfected clone N21 was inhibited by 70% relative to the empty vector-transfected cells.

Flow cytometric analysis was used to assess the effect of p16 on the cell cycle of the transfected cells (Figure 3). No significant difference in cell cycle distributions were detected in parental and empty vector-transfected cells. In contrast, the proportion of cells in the S phase were reduced by more than four times in clone N21 (5.8%) when compared with those in the vector-transfected (25.2%) and parental cells (30.5%). A corresponding increase in cell numbers in G1 phase by 22% was also observed in N21 cells (88.8%) comparing with those in the controls (66.3% and 66.4%). Our results indicate that restoration of functional p16 in NPC cells suppressed cell growth by arresting cells in the G1 phase.

### Anchorage-independent growth and tumorigenicity assays

We analysed the anchorage-independent growth transforming potential of transfected cells by their ability to form colonies in soft agar. The number and size of the colonies were comparable in parental and empty vector-transfected cells. However, *p16*-transfected clone N21 had reduced ability to grow in soft agar and the colonies formed were much smaller (16–25 cells per colony) than the colonies (> 200 cells per colony) formed by control cells. The colony forming efficiency of the *p16*-transfected cells were about tenfold lower when compared to those of the parental and vector-transfected cells. These data indicate that replacement of the *p16* gene reduced transforming potential in NPC cells.

© 1999 Cancer Research Campaign

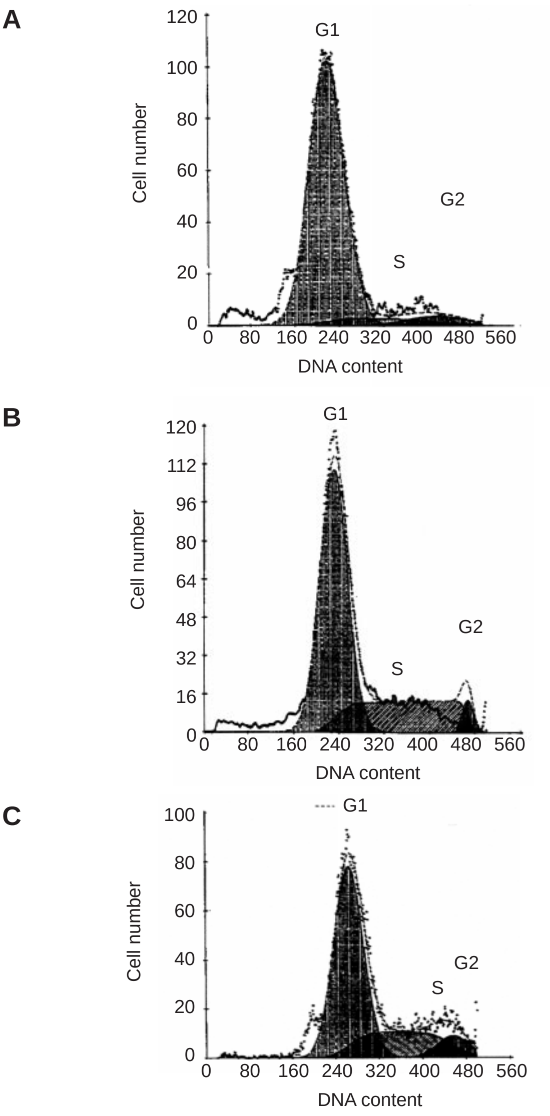

**Figure 3** Flow cytometric analyses of NPC/HK-1 cells. The p16-expressing N21 cells (**A**) displayed a shift from S to G1 phase, yielding cell cycle distribution of 88.8% G1, 5.8% S and 5.4% G2. By comparison, the parental (**B**) and vector-transfected cells (**C**) showed cell cycle profiles of 66.4% and 66.4% G1; 30.5% and 25.2% S; 3.1% and 8.5% G2 respectively

The *p16*-transfected cells as well as the parental and empty vector-transfected cells were injected into nude mice to test for their tumorigenicity. About 10 days post-inoculation, sizable tumours were observed in all nude mice injected with parental or vector-transfected cells. Table 1 depicts the growth of tumours in nude mice, 1 month after inoculation. No tumour was observed in the nude mice injected with the p16-expressing N21 cells over a period of 10 months. Our results demonstrate that re-expression of wild-type p16 in NPC cells suppressed tumorigenicity of the malignant carcinoma cells.

### DISCUSSION

It has been recognized that nasopharyngeal carcinoma, like other solid tumours, develops and progresses as a consequence of multiple genetic alterations (reviewed in Lo, 1997). Among these genetic changes, *p16* inactivation stands out in its high frequency of occurrence in this cancer. As a critical tumour suppressor, *p16* acts as an inhibitor of cdk4/cdk6 and can block the cyclin D1-dependent phosphorylation of the Rb protein. Loss of p16 protein coupled with phosphorylation of the Rb protein releases the E2F transcription factors. This signals a cell to enter into the S phase, and initiates uncontrolled cell proliferation. In this study, we have demonstrated that restoration of the wild-type *p16* activity in the *p16*-null NPC cells caused marked growth suppression and loss of tumorigenic potential. Our results indicate that loss of the *p16* gene function is an important molecular event in the tumorigenesis of NPC.

The well-characterized NPC cell line NPC/HK-1, which expresses functional Rb protein and contains no wild-type *p16* gene, was selected for the transfection study with pCMV-p16 plasmid. To investigate the tumour suppressive activity of *p16* in the NPC cells, the stable transfected cells consistently expressing exogenous wild-type p16 protein were isolated. Although many groups have reported that exogeneous p16 protein has morphological effect on several types of cancer cells (Shapiro et al, 1995; Castellano et al, 1997), we did not observe obvious morphological changes in the exogeneous p16-expressing NPC cells. It is likely that the expression of the exogenous *p16* did not alter the differentiation in these cells. The highest level of *p16* expression among these clones was found in clone N21. Study of clone N21 showed that replacement of the wild-type *p16* gene in NPC/HK-1 resulted in marked growth suppression. We have also performed apoptosis analysis and found that the number of apoptotic cells did not increase after restoration of the wild-type p16 in the NPC cells (data not shown). The result indicated that reduction of cell growth was not due to apoptosis induced by *p16*. Flow cytometry analysis of the cell cycle in N21 showed that the expression of the exogenous *p16* inhibited growth by inducing a G1 arrest of the cell cycle in these NPC cells. Our data clearly demonstrated the growth-inhibitory role of the *p16* gene in NPC. In addition, the exogenous *p16* expression in the *p16*-null NPC cells also inhibited the tumorigenic potential. By anchorage-independent colony forming assay, we found that the ability to form colonies in soft agar was highly reduced in the cells that expressed exogenous *p16*. In vivo tumorigenic study further demonstrated that these *p16*-expressing NPC cells failed to grow tumours in nude mice when compared with the control cells, for a period as long as 10 months follow-up.

Restoration of the *p16* gene into other cancer cell types has been performed previously and demonstrated similar effects on the cancer cells. The effect of replacement of the *p16* gene has been shown to be dependent on the recipient cells. Introduction of the *p16* into glioma cells (Fueyo et al, 1996) and leukaemic cells (Quesnel et al, 1996) have been shown to lead to cell growth inhibition by inducing G1 phase accumulation in the cell cycle and reduction in anchorage-independent growth ability. In melanoma cells, only cell growth inhibition and morphologic changes induction had been reported (Castellano et al, 1997). The effect of inhibiting tumorigenicity in athymic mice was also observed in the *p16*-restored colon carcinoma cells (Spillare et al, 1996) and non-small cell lung carcinoma cells (Jin et al, 1995).

Our findings have, for the first time, directly proved the critical suppressor role of the *p16* gene in the progression of NPC and suggested its potential adequacy in gene replacement therapy. As the molecular basis of NPC tumorigenesis is still not clear and only a few target genes identified for the development of gene therapy, strategies based on *p16* replacement may be one of the

*© 1999 Cancer Research Campaign* 

effective molecular approaches in the treatment of NPC. It has been reported that the reintroduction of the wild-type p53 protein in some NPC cell lines with *p53* mutations showed cytotoxic effect on the tumour cells. The high level expression of exogenous p53 protein has led to tumour cell death, mediated through apoptotic pathways in NPC cells (Li et al, 1997). The true usefulness of this gene therapy strategy remains to be tested as majority of NPC primary tumours are found to contain the wild-type *p53* gene. By mono-chromosome transfer, Cheng et al (1998) has demonstrated that chromosome 3p21.3, frequently deleted in this cancer, processes tumour suppressor function in NPC cells. The target tumour suppressor gene(s) in this region has, however, not yet been identified. Our present findings, both in vitro and in vivo, demonstrated a significant anti-tumour effect of the *p16* replacement against NPC. Some reports indicated that restoration of p16 correlated with increased radiosensitivity (Miyakoshi et al, 1997). The potential of adenovirus-mediated p16 gene transfer to NPC cells would be a logical step for the planning of our next investigation. Since the high frequency of p16 gene inactivation in NPC, the gene therapy strategies based on p16 replacement may be one of the most effective molecular approaches in the treatment of NPC.

### ACKNOWLEDGEMENTS

We thank Professor D. Sidransky of The Johns Hopkins University, USA for providing the pCMV-p16 plasmid and Mr Jesse C S Pang for suggestions and advisings the edition of this manuscript. The project was funded partly by the Research Grant Council of Hong Kong (CUHK 261/96M and CUHK 4284/98M).

### REFERENCES

Castellano M, Gabrielli BG, Hussussian CJ, Dracopoli NC and Hayward NK (1997) Restoration of CDKN2A into melanoma cells induces morphologic changes and reduction in growth rate but not anchorage-independent growth reversal. *J Invest Dermatol* **109**: 61–68

Chan FKM, Zhang J, Chen L, Shapiro DN and Winoto A (1995) Indentification of human/mouse p19, a novel cdk4/cdk6 inhibitor with homology to p16$^{ink4}$. *Mol Cell Biol* **15**: 2682–2688

Cheng RYS, Lo KW, Huang DP and Tsao SW (1997) Loss of heterozygosity on chromosome 14 in primary nasopharyngeal carcinoma. *Int J Oncol* **10**: 1047–1050

Cheng Y, Poulos NE, Lung ML, Hampton G, Ou B, Lerman MI and Stanbridge EJ (1998) Functional evidence for a nasopharyngeal carcinoma tumor suppressor gene that maps at chromosome 3p21.3. *Proc Natl Acad Sci USA* **95**: 3042–3047

Fueyo J, Gomez-Manzano C, Yung WKA, Clayman GL, Liu TJ, Bruner J, Levin VA and Kyritsis AP (1996) Adenovirus-mediated p16/CDKN2 gene transfer induces growth arrest and modifies the transformed phenotype of glioma cells. *Oncogene* **12**: 103–110

Gulley ML, Nicholls JM, Schneider BG, Amin MB, Ro JY and Geradts J (1998) Nasopharyngeal carcinomas frequently lack the p16/MTS1 tumor suppressor protein but consistently express the retinoblastoma gene product. *Am J Pathol* **152**: 865–869

Hannon GJ and Beach D (1994) p15$^{INK4b}$ is a potential effector of cell cycle arrest mediated by TGF-β. *Nature (Lond)* **371**: 257–261

Hinds PS, Mittnacht S, Dulic V, Arnold A, Reed SI and Weinberg RA (1992) Regulation of retinoblastoma protein functions by ectopic expression of human cyclins. *Cell* **70**: 993–1006

Huang DP, Ho JHC, Poon YF, Chew EC, Saw D, Liu M, Li CL, Mak LS, Lai SH and Lau EH (1980) Establishment of a cell line (NPC/HK1) from a differentiated squamous carcinomas of the nasopharynx. *Int J Cancer* **26**: 127–132

Huang DP, Lo KW, Choi PHK, Ng AYT, Tsao SY, Yiu GKC and Lee JC (1991) Loss of heterozygosity on the short arm of chromosome 3 in nasopharyngeal carcinoma. *Cancer Genet Cytogenet* **54**: 91–99

Huang DP, Lo KW, van Hasselt CA, Woo JK, Choi PH, Leung SF, Cheung ST, Cairns O, Sidransky D and Lee JC (1994) A region of homozygous deletion on chromosome 9p21–22 in primary nasopharyngeal carcinoma. *Cancer Res* **54**: 4003–4006

Hui ABY, Lo KW, Leung SF, Choi PH, Fong Y, Lee JC and Huang DP (1996) Loss of heterozygosity on the long arm of chromosome 11 in nasopharyngeal carcinoma. *Cancer Res* **56**: 3225–3229

Jin X, Nguyen D, Zhang WW, Kyritsis AP and Roth JA (1995) Cell cycle arrest and inhibition of tumor cell proliferation by the p16$^{INK4}$ gene mediated by an adenovirus vector. *Cancer Res* **55**: 3250–3253

Kamb A, Gruis A, Weaver-Feldhaus J, Liu QY, Harshman K, Tavtigian SV, Stockert E, Day III RS, Johnson BE and Skolnick MH (1994) A cell cycle regulator potentially involved in genesis of many tumor types. *Science* **264**: 436–439

Kato JY, Matsushime H, Hiebert SW, Ewen ME and Sherr CJ (1993) Direct binding of cyclin D to the retinoblastoma gene product (pRb) and pRb phosphorylation by the cyclin D-dependent kinase CDK4. *Gene Dev* **7**: 331–342

Li JH, Li P, Klamut H and Liu FF (1997) Cytotoxic effects of ad5CMV-*p53* expression in two human nasopharyngeal carcinoma cell lines. *Clin Cancer Res* **3**: 507–514

Liggett WH Jr, Sewell DA, Rocco J, Ahrendt SA, Koch W and Sidransky D (1996) p16 and p16 are potent growth suppressors of head and neck squamous carcinoma cells in vitro. *Cancer Res* **56**: 4119–4123

Lo KW, Huang DP and Lau KM (1995) p16 gene alterations in nasopharyngeal carcinoma. *Cancer Res* **55**: 2039–2043

Lo KW, Cheung ST, Leung SF, van Hasselt A, Tsang YS, Mak KF, Chung YF, Woo JKS, Lee JCK and Huang DP (1996) Hypermethylation of the p16 gene in nasopharyngeal carcinoma. *Cancer Res* **56**: 2721–2725

Lo KW, Huang DP and Lee JCK (1997) Genetic changes in nasopharyneal carcinoma (NPC). *Chinese Med J (Beijing)* **110**: 548–559

Lu QL, Elia G, Lucas S and Thoma JA (1993) Bcl-2 proto-oncogene expression in Epstein-Barr-virus-associated nasopharyngeal carcinoma. *Int J Cancer* **53**: 29–35

Miyakoshi J, Kitagawa K, Yamagishi N, Ohtsu S, Day RS III and Takebe H (1997) Increased radiosensitivity of p16 gene-deleted human glioma cells after transfection with wild-type p16 gene. *Jpn J Cancer Res* **88**: 34–38

Nobori T, Miura K, Wu DJ, Lois A, Takabaryashi K and Carson DA (1994) Deletions of the cyclin-dependent kinase-4 inhibitor gene in multiple human cancers. *Nature (Lond)* **368**: 753–756

Pagano M, Pepper R, Lukas J, Baldin V, Ansorge W, Bartek J and Draetta G (1993) Regulation of the cell cycle by the cdk2 protein kinase in cultured human fibroblasts. *J Cell Biol* **121**: 110–111

Porter MJ, Field JK, Leung SF, Lo D, Lee JC, Spandidos DA and van Hasselt CA (1994) The detection of the c-myc and ras oncogenes in nasopharyngeal carcinoma by immunohistochemistry. *Acta Otolaryngol* **114**: 105–109

Quesnel B, Preudhomme C, Lepelley P, Hetuin D, Vanrumbeke M, Bauters F, Velu T and Fenaux P (1996) Transfer of p16$^{inka}$/CDKN2 gene in leukaemic cell lines inhibits cell proliferation. *Br J Haematol* **95**: 291–298

Spillare E, Okamoto A, Hagiwara K, Demetrick DJ, Serrano M, Beach D and Harris CC (1996) Suppression of growth in vitro and tumorigenicity in vivo of human carcinoma cell lines by transfected p16$^{INK4}$. *Mol Carcinogen* **16**: 53–60

Spruck III CH, Tsai YC, Huang DP, Yang AS, Rideout III WM, Gonzalez-Zulueta M, Choi P, Lo KW, Yu MC and Jones PA (1992) Absence of p53 gene mutations in primary nasopharyngeal carcinomas. *Cancer Res* **52**: 4787–4790

 *© 1999 Cancer Research Campaign*

## References

[PDF_00466] Castellano M., Gabrielli B. G., Hussussian C. J., Dracopoli N. C., Hayward N. K. (1997). Restoration of CDKN2A into melanoma cells induces morphologic changes and reduction in growth rate but not anchorage-independent growth reversal.. J Invest Dermatol.

[PDF_00470] Chan F. K., Zhang J., Cheng L., Shapiro D. N., Winoto A. (1995). Identification of human and mouse p19, a novel CDK4 and CDK6 inhibitor with homology to p16ink4.. Mol Cell Biol.

[PDF_00477] Cheng Y., Poulos N. E., Lung M. L., Hampton G., Ou B., Lerman M. I., Stanbridge E. J. (1998). Functional evidence for a nasopharyngeal carcinoma tumor suppressor gene that maps at chromosome 3p21.3.. Proc Natl Acad Sci U S A.

[PDF_00480] Fueyo J., Gomez-Manzano C., Yung W. K., Clayman G. L., Liu T. J., Bruner J., Levin V. A., Kyritsis A. P. (1996). Adenovirus-mediated p16/CDKN2 gene transfer induces growth arrest and modifies the transformed phenotype of glioma cells.. Oncogene.

[PDF_00484] Gulley M. L., Nicholls J. M., Schneider B. G., Amin M. B., Ro J. Y., Geradts J. (1998). Nasopharyngeal carcinomas frequently lack the p16/MTS1 tumor suppressor protein but consistently express the retinoblastoma gene product.. Am J Pathol.

[PDF_00488] Hannon G. J., Beach D. (1994). p15INK4B is a potential effector of TGF-beta-induced cell cycle arrest.. Nature.

[PDF_00491] Hinds P. W., Mittnacht S., Dulic V., Arnold A., Reed S. I., Weinberg R. A. (1992). Regulation of retinoblastoma protein functions by ectopic expression of human cyclins.. Cell.

[PDF_00494] Huang D. P., Ho J. H., Poon Y. F., Chew E. C., Saw D., Lui M., Li C. L., Mak L. S., Lai S. H., Lau W. H. (1980). Establishment of a cell line (NPC/HK1) from a differentiated squamous carcinoma of the nasopharynx.. Int J Cancer.

[PDF_00498] Huang D. P., Lo K. W., Choi P. H., Ng A. Y., Tsao S. Y., Yiu G. K., Lee J. C. (1991). Loss of heterozygosity on the short arm of chromosome 3 in nasopharyngeal carcinoma.. Cancer Genet Cytogenet.

[PDF_00501] Huang D. P., Lo K. W., van Hasselt C. A., Woo J. K., Choi P. H., Leung S. F., Cheung S. T., Cairns P., Sidransky D., Lee J. C. (1994). A region of homozygous deletion on chromosome 9p21-22 in primary nasopharyngeal carcinoma.. Cancer Res.

[PDF_00505] Hui A. B., Lo K. W., Leung S. F., Choi P. H., Fong Y., Lee J. C., Huang D. P. (1996). Loss of heterozygosity on the long arm of chromosome 11 in nasopharyngeal carcinoma.. Cancer Res.

[PDF_00508] Jin X., Nguyen D., Zhang W. W., Kyritsis A. P., Roth J. A. (1995). Cell cycle arrest and inhibition of tumor cell proliferation by the p16INK4 gene mediated by an adenovirus vector.. Cancer Res.

[PDF_00512] Kamb A., Gruis N. A., Weaver-Feldhaus J., Liu Q., Harshman K., Tavtigian S. V., Stockert E., Day R. S., Johnson B. E., Skolnick M. H. (1994). A cell cycle regulator potentially involved in genesis of many tumor types.. Science.

[PDF_00515] Kato J., Matsushime H., Hiebert S. W., Ewen M. E., Sherr C. J. (1993). Direct binding of cyclin D to the retinoblastoma gene product (pRb) and pRb phosphorylation by the cyclin D-dependent kinase CDK4.. Genes Dev.

[PDF_00518] Li J. H., Li P., Klamut H., Liu F. F. (1997). Cytotoxic effects of Ad5CMV-p53 expression in two human nasopharyngeal carcinoma cell lines.. Clin Cancer Res.

[PDF_00521] Liggett W. H., Sewell D. A., Rocco J., Ahrendt S. A., Koch W., Sidransky D. (1996). p16 and p16 beta are potent growth suppressors of head and neck squamous carcinoma cells in vitro.. Cancer Res.

[PDF_00526] Lo K. W., Cheung S. T., Leung S. F., van Hasselt A., Tsang Y. S., Mak K. F., Chung Y. F., Woo J. K., Lee J. C., Huang D. P. (1996). Hypermethylation of the p16 gene in nasopharyngeal carcinoma.. Cancer Res.

[PDF_00524] Lo K. W., Huang D. P., Lau K. M. (1995). p16 gene alterations in nasopharyngeal carcinoma.. Cancer Res.

[PDF_00529] Lo K., Huang P. W., Lee C. K. (1997). Genetic changes in nasopharyngeal carcinoma.. Chin Med J (Engl).

[PDF_00531] Lu Q. L., Elia G., Lucas S., Thomas J. A. (1993). Bcl-2 proto-oncogene expression in Epstein-Barr-virus-associated nasopharyngeal carcinoma.. Int J Cancer.

[PDF_00534] Miyakoshi J., Kitagawa K., Yamagishi N., Ohtsu S., Day R. S., Takebe H. (1997). Increased radiosensitivity of p16 gene-deleted human glioma cells after transfection with wild-type p16 gene.. Jpn J Cancer Res.

[PDF_00537] Nobori T., Miura K., Wu D. J., Lois A., Takabayashi K., Carson D. A. (1994). Deletions of the cyclin-dependent kinase-4 inhibitor gene in multiple human cancers.. Nature.

[PDF_00540] Pagano M., Pepperkok R., Lukas J., Baldin V., Ansorge W., Bartek J., Draetta G. (1993). Regulation of the cell cycle by the cdk2 protein kinase in cultured human fibroblasts.. J Cell Biol.

[PDF_00543] Porter M. J., Field J. K., Leung S. F., Lo D., Lee J. C., Spandidos D. A., van Hasselt C. A. (1994). The detection of the c-myc and ras oncogenes in nasopharyngeal carcinoma by immunohistochemistry.. Acta Otolaryngol.

[PDF_00546] Quesnel B., Preudhomme C., Lepelley P., Hetuin D., Vanrumbeke M., Bauters F., Velu T., Fenaux P. (1996). Transfer of p16inka/CDKN2 gene in leukaemic cell lines inhibits cell proliferation.. Br J Haematol.

[PDF_00550] Spillare E. A., Okamoto A., Hagiwara K., Demetrick D. J., Serrano M., Beach D., Harris C. C. (1996). Suppression of growth in vitro and tumorigenicity in vivo of human carcinoma cell lines by transfected p16INK4.. Mol Carcinog.

[PDF_00554] Spruck C. H., Tsai Y. C., Huang D. P., Yang A. S., Rideout W. M., Gonzalez-Zulueta M., Choi P., Lo K. W., Yu M. C., Jones P. A. (1992). Absence of p53 gene mutations in primary nasopharyngeal carcinomas.. Cancer Res.

